# 15-Deoxy-Δ^12,14^-prostaglandin J2 promotes phosphorylation of eukaryotic initiation factor 2α and activates the integrated stress response

**DOI:** 10.1074/jbc.RA118.007138

**Published:** 2019-02-05

**Authors:** Devin Tauber, Roy Parker

**Affiliations:** From the ‡ Department of Biochemistry, University of Colorado, Boulder, Colorado 80309 and; the § Howard Hughes Medical Institute, Chevy Chase, Maryland 20815-6789

**Keywords:** prostaglandin, eukaryotic initiation factor 2 (eIF2), stress granule, stress response, neuroinflammation, proteasome

## Abstract

Stress granules (SGs) are cytoplasmic RNA–protein aggregates formed in response to inhibition of translation initiation. SGs contribute to the stress response and are implicated in a variety of diseases, including cancer and some forms of neurodegeneration. Neurodegenerative diseases often involve chronic phosphorylation of eukaryotic initiation factor 2α (eIF2α), with deletions of eIF2α kinases or treatment with eIF2α kinase inhibitors being protective in some animal models of disease. However, how and why the integrated stress response (ISR) is activated in different forms of neurodegeneration remains unclear. Because neuroinflammation is common to many neurodegenerative diseases, we hypothesized that inflammatory factors contribute to ISR activation in a cell-nonautonomous manner. Using fluorescence microscopy and immunoblotting, we show here that the endogenously produced product of inflammation, 15-deoxy-Δ^12,14^-prostaglandin J2 (15-d-PGJ2), triggers eIF2α phosphorylation, thereby activating the ISR, repressing bulk translation, and triggering SG formation. Our findings define a mechanism by which inflammation activates the ISR in a cell-nonautonomous manner and suggest that inhibition of 15-d-PGJ2 production might be a useful therapeutic strategy in some neuroinflammatory contexts.

## Introduction

The ISR^2^ is an important intracellular signaling pathway cells use to respond to a wide variety of stressors (1, 2). When cells undergo oxidative, proteotoxic, or nutrient deprivation stress, one or more of four eIF2α kinases is activated to phosphorylate eIF2α (2). P-eIF2α serves as a competitive inhibitor for the eIF2 guanine exchange factor eIF2B so that, when eIF2α phosphorylation occurs, the levels of GTP-eIF2 decrease, leading to bulk translation repression and preferential translation of stress response mRNAs (2–4). mRNAs preferentially translated under stress often contain u (upstream) ORFs or IRESs (internal ribosomal entry site) and include stress response proteins such as mRNAs for the ATF transcription factor family, cytokines/chemokines, or the pro-inflammatory enzyme COX-2 (2, 3, 5, 6). The production of these proteins allows both cell- and noncell-autonomous communication to resolve cellular stress or propagate inflammation (2, 5–7).

When translation initiation is strongly inhibited, mRNPs accumulate in RNA–protein granules in the cytosol, referred to as stress granules (SGs) (3, 8–10). *In vivo*, SGs are most commonly formed because of ISR activation and eIF2α phosphorylation (1, 2, 5, 11–14) but can also form because of inhibition of eIF4A function (9). Strikingly, the ISR, which, although beneficial acutely, is chronically activated in many neurodegenerative diseases (1–2, 4, 12, 14–17). Chronic activation of the ISR can lead to cell death, and ISR inhibitors such as ISRIB, PKR inhibitor (C16, PKRi), and PERK inhibitor (GSK2606414, PERKi) have been shown to be neuroprotective in mouse models of disease (2, 4, 12, 15, 18, 19). This leads to the hypothesis that chronic eIF2α phosphorylation contributes to disease progression concomitant with chronic neuroinflammation, but the mechanisms by which products of inflammation trigger phosphorylation of eIF2α are poorly understood (2, 5, 6).

Prostaglandins are a class of neuroinflammatory molecules that are synthesized after environmental insults to the brain and are chronically secreted under many forms of neurodegenerative disease (20–24). The most abundant prostaglandin in the brain, PGD2, can undergo spontaneous dehydration reactions to form the J2 class of prostaglandins (20, 21, 24, 25). These molecules are characterized by a cyclopentanone moiety and α,β-unsaturated double bonds, serving as electrophiles for covalent modification of cysteine residues on target proteins (20, 21, 26). The most reactive of the J2 prostaglandins, 15-d-PGJ2, has been shown to promote SG formation in cells, making it one of a few known endogenously produced inducers of SGs (10, 27). 15-d-PGJ2 is known to covalently modify eIF4A (10, 26), and it has been concluded that 15-d-PGJ2 modification of eIF4A inhibits translation initiation and leads to SG formation (10). However, 15-d-PGJ2 has also been shown to cause eIF2α phosphorylation (28).

In the course of analyzing the cellular response to 15-d-PGJ2, we observed that 15-d-PGJ2 represses bulk translation and triggers an ISR by causing eIF2α phosphorylation. This leads to the hypothesis that 15-d-PGJ2 is a product of inflammation that can chronically activate the ISR, contribute to cell death, and potentially drive neurodegenerative disease progression in a cell-nonautonomous manner.

## Results

### SGs triggered by 15-d-PGJ2 display similar kinetics to SGs induced by NaAsO_2_ and are dismantled upon treatment with ISRIB

15-d-PGJ2 has been suggested to inhibit eIF4A, although it was originally observed that this prostaglandin can induce P-eIF2α (10, 28). To clarify this issue and determine whether 15-d-PGJ2 triggered SGs by inhibition of eIF4A or by promoting eIF2α phosphorylation, we first examined the effect of ISRIB on SGs formed after 15-d-PGJ2 exposure. The small-molecule ISRIB enhances eIF2B function and negates the inhibitory effects of P-eIF2α (12, 29). Thus, ISRIB prevents SG formation in response to eIF2α phosphorylation and promotes the disassembly of pre-existing eIF2α phosphorylation-dependent SGs (4). However, ISRIB has no effects on SG formation via inhibition of eIF4A, which blocks translation upstream of eIF2α function (4). Therefore, if 15-d-PGJ2 acts by inhibiting eIF4A, then SG formation triggered by 15-d-PGJ2 should be resistant to ISRIB addition. In contrast, if 15-d-PGJ2 acts through eIF2α phosphorylation, then SGs formed by 15-d-PGJ2 should be sensitive to ISRIB.

**Figure 1.**
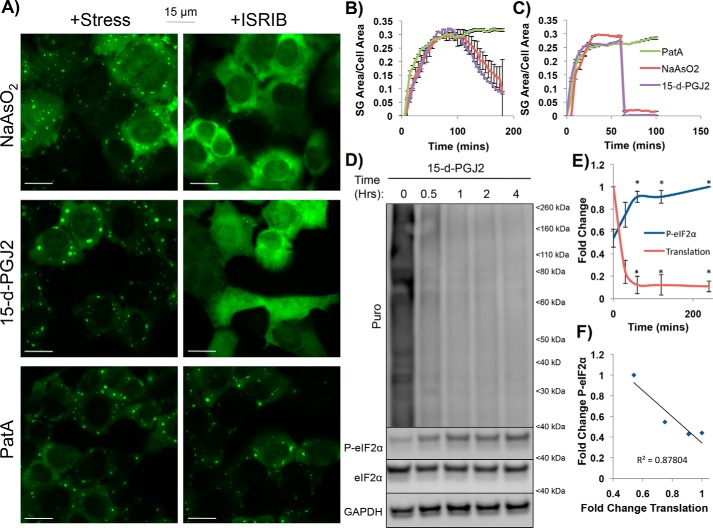
**ISRIB disrupts 15-d-PGJ2–induced stress granules, and translational shutoff coincides with eIF2α phosphorylation in GFP-G3BP1 U-2 OS cells.** *A*, U-2 OS cells stably expressing the SG marker GFP-G3BP1 were treated with NaAsO_2_ (100 μm), PGJ2 (50 μm), or PatA (100 nm) for 1 h and imaged (*left panels*). ISRIB (5 nm) was then added, and cells were imaged 5 min later (*right panels*) (*scale bars* represent 15 μm). *B*, SG induction kinetics were assessed by quantifying SG area per cell area over time following stress by NaAsO_2_ (100 μm), 15-d-PGJ2 (50 μm), or PatA (100 nm). Shown is the average (± S.D.) SG area (μm^2^) of SGs from an entire frame from images collected in live U-2 OS cells every 5 min for 200 min (*n* = 3). *C*, quantification of ISRIB addition SG kinetics from *A*, depicting SG area per cell area per time; 15-d-PGJ2 and NaAsO_2_ SGs disappear rapidly whereas PatA SGs do not (*n* = 3). *D*, immunoblot depicting puromycin incorporation into nascent peptides as a measure of translation in conjunction with P-eIF2α induction over a period of 4 h after treatment with 10 μm 15-d-PGJ2. Puromycin incorporation decreases, whereas P-eIF2α increases, after 15-d-PGJ2 addition. *E*, quantification of *D* with normalization to the 0-min time point. Translational reduction and P-eIF2α induction display inverse kinetics in relation to each other, with translational reduction plateauing when P-eIF2α levels reach a steady-state maximum after 15-d-PGJ2 stress. *, *p* < 0.05; unpaired Student's *t* test; results are displayed as the mean ± S.D.; *n* = 3. *F*, correlation plot between P-eIF2α and translation from -fold changes depicted in *E*. Translation and P-eIF2α negatively correlate after 15-d-PGJ2 addition, suggesting that the two are interrelated (R^2^ = 0.878).

We used cells expressing a stable GFP-G3BP1 fusion protein to track the kinetics of SG assembly (quantified by SG area per cell area) over time. We compared the kinetics of SG assembly upon treatment with 15-d-PGJ2 to NaAsO_2_-induced SGs, (which form upon eIF2α phosphorylation) and pateamine A (PatA, which triggers SG formation by inhibiting eIF4A) (9). Without any ISRIB addition, NaAsO_2_- and 15-d-PGJ2-induced SG kinetics closely resembled each other, with an SG growth phase that reached its maximum around 60 min and an eventual size reduction phase after about 100 min (Fig. 1*B*). Conversely, PatA-induced SGs solely displayed a growth phase, which, after about 60 min, resulted in SG area stagnation (Fig. 1*B*). PatA-induced granules have no known negative feedback loop, and after 200 min, the granules still had no reduction in area.

An important result was that, when cells were treated with 5 nm ISRIB, SGs that formed because of NaAsO_2_ or 15-d-PGJ2 stresses disappeared within 5 min, whereas PatA SGs were resistant to ISRIB treatment (Fig. 1, *A* and *C*). This suggests that 15-d-PGJ2 inhibits translation and promotes SG formation through eIF2α phosphorylation and not through inhibition of eIF4A function.

### 15-d-PGJ2–treated cells have elevated levels of P-eIF2α

Because ISRIB was able to inhibit SGs in 15-d-PGJ2–treated cells, we tested whether global translation suppression in 15-d-PGJ2–treated cells was coupled to eIF2α phosphorylation. If global translation suppression occurred simultaneously (and therefore correlated) with eIF2α phosphorylation, then this would suggest that P-eIF2α may be involved in mediating translational attenuation and SG formation. We treated U-2 OS cells with 15-d-PGJ2 over a period of 4 h, and cells were briefly treated with puromycin in a 5-min pulse before lysates were collected to monitor both P-eIF2α and puromycin incorporation into nascent peptides as a measure of bulk translation activity. We then used immunoblotting to quantify P-eIF2α and translational kinetics during the cellular response to 15-d-PGJ2 treatment.

Compared with unstressed cells, cells treated with 15-d-PGJ2 showed about 2-fold more eIF2α phosphorylation, which peaked and plateaued at about 1 h. Additionally, 15-d-PGJ2–treated cells showed a reduction in translation that plateaued simultaneously with P-eIF2α (Fig. 1, *C* and *D*). Using a correlation plot to examine how related these two events might be, we found a substantial correlation between the kinetics of eIF2α phosphorylation and translational attenuation (R^2^ = 0.876), suggesting that the two events are coupled (Fig. 1*E*).

### ISRIB partially restores translation in 15-d-PGJ2–treated cells

In principle, ISRIB could trigger SG disassembly by restoring translation in 15-d-PGJ2–treated cells or by an unrelated mechanism. To examine whether translation was being restored upon ISRIB treatment, U-2 OS cells stably expressing GFP-G3BP1 were treated with NaAsO_2_ or 15-d-PGJ2, ISRIB was added 1 h post-stress, and then cells were pulsed with puromycin for 5 min. Subsequently, the cells were fixed and stained with an anti-puromycin antibody (α-puro), or lysates were collected for immunoblotting.

**Figure 2.**
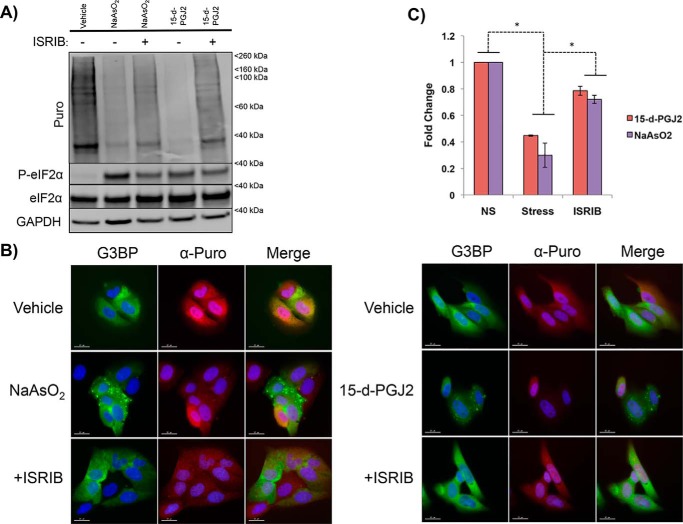
**ISRIB partially restores translation in 15-d-PGJ2–treated U-2 OS cells.** *A*, immunoblot depicting puromycin incorporation as a marker for translation to examine the effects of ISRIB on translation and P-eIF2α. U-2 OS cells were stressed with NaAsO_2_ (100 μm) or 15-d-PGJ2 (10 μm) for an hour and subsequently treated with ISRIB (5 nm) for 5 min before puromycin pulsing. Notice that P-eIF2α is unchanged, whereas translation is partially restored, by ISRIB for both NaAsO_2_ and 15-d-PGJ2. *B*, images depicting α-puro fluorescence intensity in U-2 OS cells exposed to NaAsO_2_ or 15-d-PGJ2 ± ISRIB addition to confirm the results from *A*. U-2 OS cells were subjected to the same conditions as in *A* but fixed and stained for immunofluorescence. α-Puro fluorescence intensity drops an hour after being exposed to NaAsO_2_ and 15-d-PGJ2 but is partially restored after ISRIB addition. Note the single cell in both NaAsO_2_ and 15-d-PGJ2 frames displaying high α-puro fluorescence intensity while not containing SGs. Presumably, translation is not shut off in those cells, and therefore they do not contain SGs (*scale bars* represent 15 μm). *C*, quantification of fluorescence intensities from *B*, depicting an increase in translation after ISRIB addition for both NaAsO_2_ and 15-d-PGJ2. *, *p* < 0.05; unpaired Student's *t* test; results are displayed as the mean ± S.D.; *n* = 3).

As expected, we observed that both NaAsO_2_- and 15-d-PGJ2–treated cells showed decreased translation, as assessed by puromycin incorporation detected via immunoblotting (Fig. 2*A*). In addition, ISRIB rescued translation in cells treated with either NaAsO_2_ or 15-d-PGJ2 (Fig. 2*A*). Similar results were observed by examining individual cells, where we determined that treatment with NaAsO_2_ or 15-d-PGJ2 reduced the α-puromycin fluorescence signal (Fig. 2, *B* and *C*). Moreover, upon ISRIB treatment, α-puromycin fluorescence signal quantification showed a partial restoration of translation in both NaAsO_2_- and 15-d-PGJ2–treated cells (Fig. 2, *B* and *C*). These data suggest that ISRIB is not functioning with off-target effects in the case of 15-d-PGJ2–induced SGs because it is restoring translation in a similar manner as NaAsO_2_-induced SGs. These results are consistent with 15-d-PGJ2 inhibiting translation through a mechanism dependent on P-eIF2α.

### eIF2α phosphorylation is required for 15-d-PGJ2–induced stress granules

**Figure 3.**
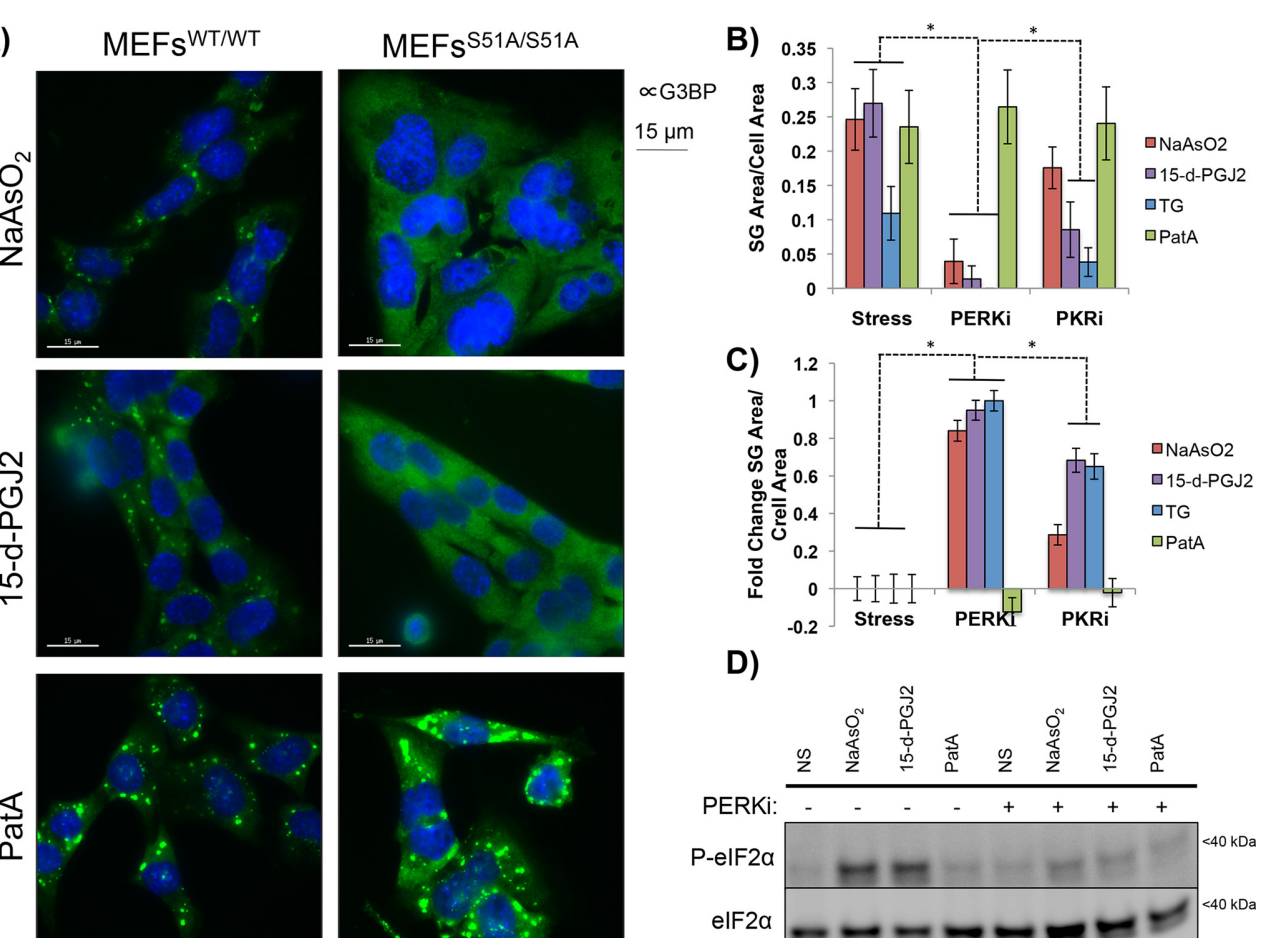
**15-d-PGJ2 requires eIF2α phosphorylation for SG induction and is dependent on an eIF2α kinase.** *A*, MEFs^WT/WT^ or MEFs^S51A/S51A^ were stressed with NaAsO_2_ (100 μm), 15-d-PGJ2 (50 μm), or PatA (100 nm) for an hour and fixed and stained for G3BP immunofluorescence. SGs form under all conditions in MEFs^WT/WT^, but only PatA SGs form in MEFs^S51A/S51A^, indicating that P-eIF2α is required for SGs driven by 15-d-PGJ2 (*scale bars* represent 15 μm). *B*, U-2 OS cells expressing GFP-G3BP were preincubated with 1 μm PERKi or PKRi for 15 min and stressed with NaAsO_2_ (100 μm), 15-d-PGJ2 (50 μm), TG (500 nm), or PatA (100 nm) for 1 h and fixed. Quantification of SG area per cell area was performed to examine how PERKi or PKRi preincubation affects SG formation for the various stressors. Although inconclusive regarding a responsible eIF2αK for 15-d-PGJ2–induced SGs, both inhibitors prevent 15-d-PGJ2 SGs, indicating that an eIF2αK is required. *, *p* < 0.05; unpaired Student's *t* test; results are displayed as the mean ± S.D.; *n* = 3). *C*, differential graph depicting magnitudes of SG inhibition normalized to conditions without eIF2αK inhibitors present. PERKi prevents SG formation by all stressors except PatA and is a pan-ISR inhibitor. *, *p* < 0.05; unpaired Student's *t* test; results are displayed as the mean ± S.D.; *n* = 3). *D*, U-2 OS cells were treated under the same conditions outlined in *B* but were lysed for immunoblotting to examine how PERKi affected 15-d-PGJ2–induced P-eIF2α. eIF2α phosphorylation is prevented by PERKi in both NaAsO_2_- and 15-d-PGJ2–treated cells, consistent with P-eIF2α being required for 15-d-PGJ2 SG formation.

The above results were consistent with a model wherein 15-d-PGJ2 triggers SG formation by enhancing eIF2α phosphorylation. To directly test this model, we examined whether 15-d-PGJ2 could trigger SG formation in mouse embryonic fibroblasts (MEFs) that harbor mutations in the phosphorylation site in eIF2α (homozygous for S51A mutations). WT MEFs (MEFs^WT/WT^) or MEFs expressing eIF2S1^S51A/S51A^ (MEFs^S51A/S51A^) were treated for 1 h with NaAsO_2_, 15-d-PGJ2, or PatA, and immunofluorescence microscopy was performed to image G3BP as a marker for SGs. As expected for PatA-treated MEFs, SGs could form in both cell types, whereas SGs could only form in MEFs^WT/WT^ treated with NaAsO_2_. Importantly, MEFs^S51A/S51A^ could not form SGs upon treatment with 15-d-PGJ2 (Fig. 3*A*). These data demonstrate that 15-d-PGJ2 requires phosphorylation of eIF2α to trigger SG formation.

### Multiple eIF2α kinases are activated by 15-d-PGJ2

Mammalian cells contain four eIF2α kinases (eIF2αKs): HRI, PERK, PKR, and GCN2 (2, 9). However, P-eIF2α levels can also increase upon inhibition of the phosphatase PP1, which forms a complex with the stress-induced GADD34 protein to dephosphorylate P-eIF2α (17). As a first step to determine whether a specific or multiple kinases are activated by 15-d-PGJ2, we examined how small-molecule inhibitors for eIF2αKs affected 15-d-PGJ2 induction of SGs. To test whether these inhibitors had an effect, U-2 OS GFP-G3BP1–expressing cells were pretreated with PKRi or PERKi for 15 min and then treated with either NaAsO_2_ (known to activate HRI), 15-d-PGJ2, thapsigargin (TG, known to activate PERK), or PatA (inhibits eIF4A) (9). Cells were fixed and imaged, and SG area per cell area was quantified in the presence of either inhibitor (Fig. S1).

We observed that both the SG area and cell area of 15-d-PGJ2 and TG SGs were reduced after pretreatment with PKRi, suggesting that either PKRi might inhibit TG-induced activation of PERK or that TG activates PKR (Fig. 3, *B* and *C*, and Fig. S1). We next monitored the SG response of our panel of stressors in the presence of PERKi and found that all stressors except PatA had a reduced SG area per cell area (Fig. 3, *B* and *C*, and Fig. S1). Immunoblotting for P-eIF2α showed that PERKi prevented P-eIF2α for both NaAsO_2_ and 15-d-PGJ2 (Fig. 3*D*). These data, although inconclusive regarding the responsible kinase for eIF2α phosphorylation in the presence of 15-d-PGJ2, confirm that an eIF2αK is responsible for SG induction and also suggest that PERKi may be affecting other eIF2αKs besides PERK.

**Figure 4.**
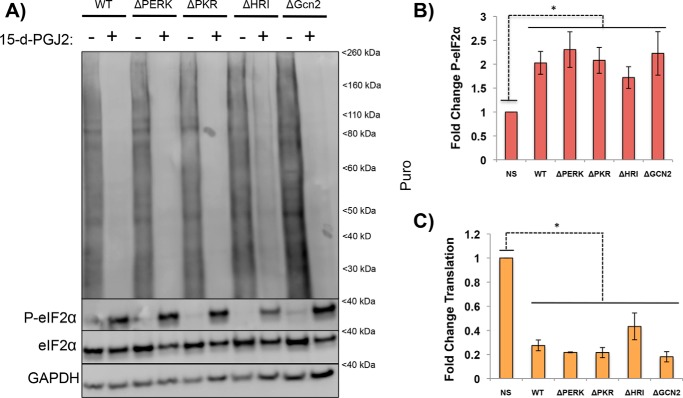
**Loss of a single eIF2αK does not prevent 15-d-PGJ2–driven P-eIF2α.** *A*, HAP1 cells that were either WT or contained a single eIF2αK deletion were stressed with 15-d-PGJ2 (10 μm) for 1 h, pulsed with puromycin for 5 min, and lysed for immunoblotting to examine how eIF2αK deletions affected 15-d-PGJ2 eIF2α phosphorylation and translation. No single eIF2αK deletion prevents translational shutoff or P-eIF2α; however, ΔHRI has a modest effect on preventing translational shutoff and eIF2α phosphorylation, similar to MG132 (Fig. S3*A*). *B*, quantification of P-eIF2α from *A*. No single kinase deletion had a significant effect on decreasing P-eIF2α, but ΔHRI had less P-eIF2α than the rest. *, *p* < 0.05; unpaired Student's *t* test; results are displayed as the mean ± S.D.; *n* = 3; *NS*, not significant. *C*, quantification of translation from *A*. No single eIF2α kinase prevents translational shutoff or significant changes from the WT, but ΔHRI had less of a decrease. *, *p* < 0.05; unpaired Student's *t* test; results are displayed as the mean ± S.D.; *n* = 3.

To more directly examine what eIF2αKs were activated by 15-d-PGJ2 treatment, we examined how 15-d-PGJ2 affected translation and eIF2α phosphorylation in haploid human cells (HAP1) that were either WT or lacking a specific eIF2αK (9). Surprisingly, we observed that no single eIF2αK deletion prevented eIF2α phosphorylation or strongly reduced translation repression in response to 15-d-PGJ2, with only the cell line lacking HRI showing any differences from WT cells (Fig. 4, *A–C*). This suggests that multiple kinases are activated by 15-d-PGJ2 and produce a combinatorial effect in phosphorylating eIF2α (Fig. 4, *B* and *C*). As controls, we reproduced earlier results showing that NaAsO_2_ translation repression depends on HRI (9) and that the 26S proteasomal inhibitor MG132 induces translation repression through multiple eIF2αKs (Fig. S3, *A* and *B*) (9). Taken together, these observations argue that 15-d-PGJ2 activates multiple eIF2αKs and that those act together to phosphorylate eIF2α, repress translation, and trigger SG formation.

### 15-d-PGJ2 does not solely activate the ISR by 26S proteasome inhibition

One possible mechanism by which 15-d-PGJ2 could activate eIF2αKs is to inhibit the 26S proteasome (30). This was suggested by earlier MS data showing that 15-d-PGJ2 covalently modifies regulatory subunits of the 26S proteasome (Fig. S3*A*) (26). Moreover, similar to 15-d-PGJ2, the proteasome inhibitor MG132 triggers eIF2α phosphorylation and translation repression through multiple eIF2αKs (9). To test whether 15-d-PGJ2 triggers the ISR analogously to MG132 by proteasome inhibition, we examined how MG132 and 15-d-PGJ2 affected the relative timing of eIF2α phosphorylation, translation repression, and accumulation of K48 polyubiquitin (poly-Ub) conjugates as detected by immunoblots with antisera against ubiquitin.

**Figure 5.**
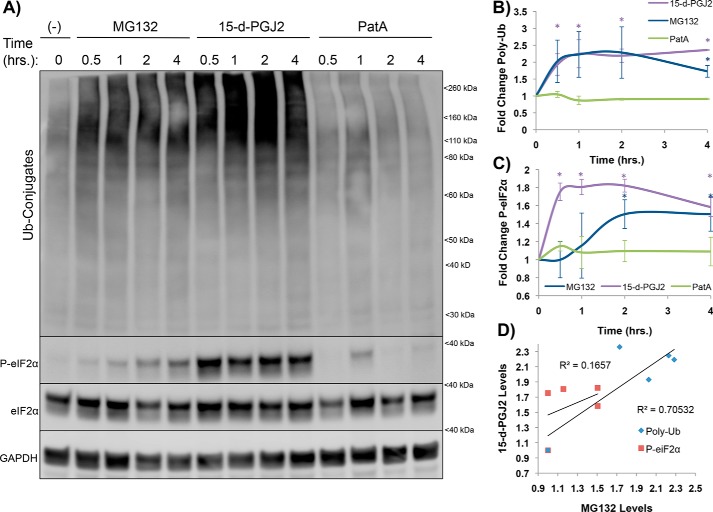
**Proteasomal inhibition precedes P-eIF2α for MG132 but coincides with P-eIF2α for 15-d-PGJ2, suggesting different mechanisms of eIF2α phosphorylation.** *A*, U-2 OS cells were stressed with MG132 (10 μm), 15-d-PGJ2 (10 μm), or PatA (100 nm) over a period of 4 h and lysed for immunoblotting to examine the kinetic relationship of K48 poly-Ub accumulation (26S inhibition) and P-eIF2α between stressors. *B*, quantification of 26S inhibition kinetics. Both MG132 and 15-d-PGJ2 display similar curves. *, *p* < 0.05; unpaired Student's *t* test; results are displayed as the mean ± S.D.; *n* = 3. *C*, quantification of P-eIF2α kinetics. 15-d-PGJ2 displays faster P-eIF2α induction than MG132, suggesting that 26S inhibition precedes proteasomal induction of P-eIF2α for 15-d-PGJ2 and that 15-d-PGJ2–induced P-eIF2α is not solely explained by 26S inhibition but could be a combinatorial effect from the proteins outlined in Fig. S2*A*. *, *p* < 0.05; unpaired Student's *t* test; results are displayed as the mean ± S.D.; *n* = 3. *D*, correlation plot between MG132- and 15-d-PGJ2–induced poly-Ub and P-eIF2α. Although poly-Ub correlates between stressors (R^2^ = 0.705), P-eIF2α does not (R^2^ = 0.166).

An important result was that, although poly-Ub accumulation was similar with MG132 or 15-d-PGJ2 treatment, eIF2α phosphorylation occurred earlier upon 15-d-PGJ2 treatment (Fig. 5, *A–C*). Because similar levels of poly-Ub conjugates are observed with 15-d-PGJ2 and MG132, but P-eIF2α occurs much earlier with 15-d-PGJ2, it strongly suggests that eIF2α phosphorylation in response to 15-d-PGJ2 is not a direct consequence of proteasome inhibition (Fig. 5*D*). It remains possible that proteasome inhibition may contribute to eIF2α phosphorylation at later time points. It is also formally possible that differential inhibition of proteasomal subunits between MG132 and 15-d-PGJ2 leads to temporal differences in activation of the ISR or that inhibition of other proteins in conjunction with the 26S proteasome causes faster phosphorylation of eIF2α by 15-d-PGJ2. Previous work demonstrated that 15-d-PGJ2 can covalently modify proteins and alter their function (26). More likely, we suggest that 15-d-PGJ2 modifies and inhibits numerous cellular proteins, such as HSP90, PP1, tRNA synthetases, 26S proteasome subunits, mitochondrial proteins, and translation initiation factors (Fig. S2*A*), that, in combination, lead to multipronged activation of the ISR (2, 26, 31, 32).

## Discussion

We present several lines of evidence showing that the prostaglandin 15-d-PGJ2 represses bulk translation and triggers SG formation through phosphorylation of eIF2α. First, we observed that SG and translation repression induced by 15-d-PGJ2 are reduced by ISRIB, which negates the effects of eIF2α phosphorylation (Figs. 1 and 2). Second, 15-d-PGJ2 fails to induce SGs in MEF^S51A/S51A^ cell lines, where the eIF2α phosphorylation site is mutated so that it is not responsive to stress-activated eIF2αKs (Fig. 3*A*). Third, SG induction by 15-d-PGJ2 is reduced by inhibitors of eIF2αKs, but eIF2α phosphorylation is present under various kinase deletion backgrounds (Figs. 3*B* and 4). Taken together, these results strongly argue that 15-d-PGJ2 triggers a cellular response that activates multiple eIF2αKs, leading to translation repression and SG induction.

In contrast to our results, 15-d-PGJ2 has also been suggested previously to inhibit translation initiation and trigger SG formation by inhibiting eIF4A function (10). Although it is clear that 15-d-PGJ2 can covalently modify eIF4A, our data strongly suggest that the major mode by which 15-d-PGJ2 represses translation is through activation of eIF2αKs. However, it remains possible that 15-d-PGJ2 modification of eIF4A contributes to translation repression and SG formation in a minor manner or might be a more prevalent mechanism for translation repression in different cell types.

An interesting implication of this work is that 15-d-PGJ2 may contribute to or be responsible for ISR activation across the spectrum of neurodegenerative diseases characterized by proteasome inhibition/poly-Ub accumulation and chronic inflammation. 15-d-PGJ2 is up-regulated in ALS and Alzheimer's disease (AD) brains (20–21, 23, 25) and can result in SG formation, poly-Ub buildup, and cell death resembling markers observed in ALS, AD, and traumatic brain injury (20, 33–35). Interestingly, COX-2 inhibitors have been shown to slow disease progression in mouse models of ALS and AD (36–39). Therefore, targeting 15-d-PGJ2 could serve as an important therapeutic approach to treating neurodegenerative diseases in which chronic ISR activation is known to play a role.

One potential measure of the utility of targeting 15-d-PGJ2 for treatment of neurodegenerative diseases in humans is the efficacy of COX-2 inhibitors in clinical trials. However, COX-2 inhibition has been met with conflicting results in clinical trials for ALS and AD (40–41). One issue with clinical trials is that drugs are administered after the onset of disease, whereas in mouse models of disease, drugs are administered near birth (36–42). Therefore, the observations that COX-2 inhibitors ameliorate disease in mouse models could be due to the timing of drug administration. Additionally, COX-2 inhibition can have many detrimental side effects in humans at doses required to efficiently inhibit prostaglandin production (40–42). If 15-d-PGJ2 is at all involved in driving neurodegeneration, then irreversible covalent modifications of translational and proteostatic factors likely build up after onset of inflammation, eventually leading to chronic ISR activation and cell death. The ISR inhibitors PKRi, PERKi, and ISRIB are showing promise for future treatment of neurodegenerative disease, and 15-d-PGJ2 is potentially one of many unknown factors that serve as a common denominator of neuroinflammation that can induce long-term and aberrant ISR activation.

## Experimental procedures

### Cell culture and drug treatments

All cell lines were grown and maintained in an incubator at 37 °C at 5% CO_2_. Cells were grown in DMEM with 10% FBS and 1% penicillin–streptomycin. For drug treatments, cells were passaged into 12-mm cell culture dishes at a final concentration 1.87 × 10^5^ cells ml^−1^ and allowed to adhere overnight. For SG induction, the medium was replaced with serum-free medium containing either NaAsO_2_ (100 μm in H_2_O, Sigma-Aldrich), 15-d-PGJ2 (10 μm in DMSO, Cayman Chemical Co.), thapsigargin (500 nm in DMSO, Sigma-Aldrich), pateamine A (100 nm in DMSO), or MG132 (10 μm in DMSO, Sigma-Aldrich), and cells were incubated at 37 °C for the allotted times indicated in each assay. For ISRIB treatment, 5 nm ISRIB (in DMSO, Sigma-Aldrich) was added after an hour of stress addition. For eIF2αK inhibitor experiments, cells were preincubated with either 1 μm PKRi (in DMSO, Sigma-Aldrich) or 1 μm PERKi (in DMSO, Sigma-Aldrich) for 15 min before stress addition. For ribopuromycinylation assays, cells were incubated at 37 °C with puromycin (10 μg ml^−1^ in H_2_O, Sigma-Aldrich) 5 min prior to fixation or lysis.

### Immunoblotting

Following drug treatment, cells were washed with 37 °C PBS and lysed with NP-40 lysis buffer (50 mm Tris-HCl (pH 8.0), 150 mm NaCl, 1% NP-40, and protease inhibitor mixture (Thermo Scientific)). Cell lysates were rocked at 4 °C for 30 min and then clarified by centrifugation (13,000 rpm for 60 s). 4× NuPAGE sample buffer was added to lysates to final concentration of 1×. Samples were boiled for 5 min at 95 °C, loaded into 4–12% BisTris NuPAGE gel, and transferred to a nitrocellulose membrane. Membranes were blocked with 5% BSA in TBS with 0.1% Tween 20 (TBST) for an hour and then incubated with primary antibody overnight at 4 °C (Table 1). Membranes were washed three times with TBST and then incubated at room temperature for 2 h in 5% BSA in TBST. Membranes were again washed three times in TBST, and antibody detection was achieved by rocking the membranes in Pierce ECL Western blotting substrate for 5 min. Chemiluminescence was visualized on an Image Quant LAS 4000 (GE Healthcare). Protein band density was quantified in ImageJ.

**Table 1:** **Antibodies used in this study**

Antigen	Company	Lot number	Dilution
P-eIF2α	CST	21	1:1000
eIF2α	CST	15	1:1000
Poly-ubiquitin	CST	16	1:1000
GAPDH	CST	4	1:1000
Puromycin	Millipore	309178	1:1000
G3BP	Abcam	GR3182998	1:1000
PABP	Abcam		1:1000
Anti-mouse HRP	CST	27	1:500
Anti-rabbit HRP	CST	33	1:500
Alexa Flour 488	Abcam	GR3202000	1:1000
Alexa Flour 594	Abcam	GR3201193	1:1000

### Immunofluorescence assays

Cells were prepared as described above but grown on glass coverslips. After drug treatments, cells were washed with warm PBS and then fixed with 4% paraformaldehyde for 15 min. Coverslips were washed with PBS and blocked with 5% BSA in PBS-T (0.1% Triton X-100) for an hour at room temperature. Coverslips were incubated with primary antibody (1:500) overnight at 4 °C in 1% BSA in PBS-T. Coverslips were then washed three times with PBS and incubated with a secondary antibody (1:1000) at room temperature for 2 h in 1% BSA in PBS-T. Coverslips were washed three times in PBS and Vectashield mounting medium containing 4′,6-diamidino-2-phenylindole for visualization of nuclei.

### Microscopy and SG quantification

For live-cell imaging, U-2 OS cells expressing GFP-G3BP1 were prepared as described above but grown in 12-mm glass-bottom dishes (Thermo Scientific). Drug treatments were added, and cells were imaged on a Nikon Ti-E spinning disk confocal microscope at 37 °C and 5% CO_2_ for the indicated time course. Images were taken every 5 min using a ×100 objective. For fixed-cell microscopy, slides were imaged on a Delta-Vision Olympus IX71 confocal microscope with a ×100 objective using softWorx software. SG area per cell area was quantified using particle finder in ImageJ. The fluorescence intensity of puromycin-labeled nascent peptides in individual cells was quantified using mean gray values in ImageJ. For all microscopy-based experiments, more than 100 cells were analyzed from three independent experimental replicates each.

## Author contributions

D. T. and R. P. conceptualization; D. T. data curation; D. T. formal analysis; D. T. and R. P. funding acquisition; D. T. and R. P. investigation; D. T. visualization; D. T. and R. P. methodology; D. T. writing-original draft; R. P. resources; R. P. supervision; R. P. project administration; R. P. writing-review and editing.

## Supplementary Material

Supporting Information

## Footnotes

2 The abbreviations used are:

ISR: integrated stress response
ISRIB: ISR inhibitor
SG: stress granule
PKRi: PKR inhibitor
PERKi: PERK inhibitor
15-d-PGJ2: 15-deoxy-Δ^12,14^-prostaglandin J2
MEF: mouse embryonic fibroblast
eIF2αK: eIF2α kinase
TG: thapsigargin
Ub: ubiquitin
AD: Alzheimer's disease
Puro: puromycin
PatA: pateamine A
OS: osteosarcoma
PKR: protein kinase R
PERK: PKR-like endoplasmic reticulum kinase
HRI: heme-regulated inhibitor
Gcn2: general control non-derepressible 2.
