# Supplementary material for: 15-Deoxy-Δ12,14-prostaglandin J2 promotes phosphorylation of eukaryotic initiation factor 2α and activates the integrated stress response
Source: J Biol Chem. 2019 Feb 5;294(16):6344–52. doi: 10.1074/jbc.RA118.007138 (PMC6484127; doi:10.1074/jbc.RA118.007138)
Supplement: Supporting Information [file supp_294_16_6344__index.html]

15-Deoxy-Δ12,14-prostaglandin J2 promotes phosphorylation of eukaryotic initiation factor 2α and activates the integrated stress response — 15-Deoxy-Δ12,14-prostaglandin J2 upregulates P-eIF2α — 15-Deoxy-Δ12,14-prostaglandin J2 promotes phosphorylation of eukaryotic initiation factor 2α and activates the integrated stress response — 15-Deoxy-Δ12,14-prostaglandin J2 up-regulates P-eIF2α — Supporting Information 

# 15-Deoxy-Δ12,14-prostaglandin J2 promotes phosphorylation of eukaryotic initiation factor 2α and activates the integrated stress response

## Supporting Information

- Supporting Information (to be published online) - Supporting figures with legends
