# Supplementary material for: 15-Deoxy-Δ12,14-prostaglandin J2 promotes phosphorylation of eukaryotic initiation factor 2α and activates the integrated stress response
Source: J Biol Chem. 2019 Feb 5;294(16):6344–52. doi: 10.1074/jbc.RA118.007138 (PMC6484127; doi:10.1074/jbc.RA118.007138)
Supplement: Supporting Information [file supp_RA118.007138_142550_1_supp_268010_plc2nf.pdf]

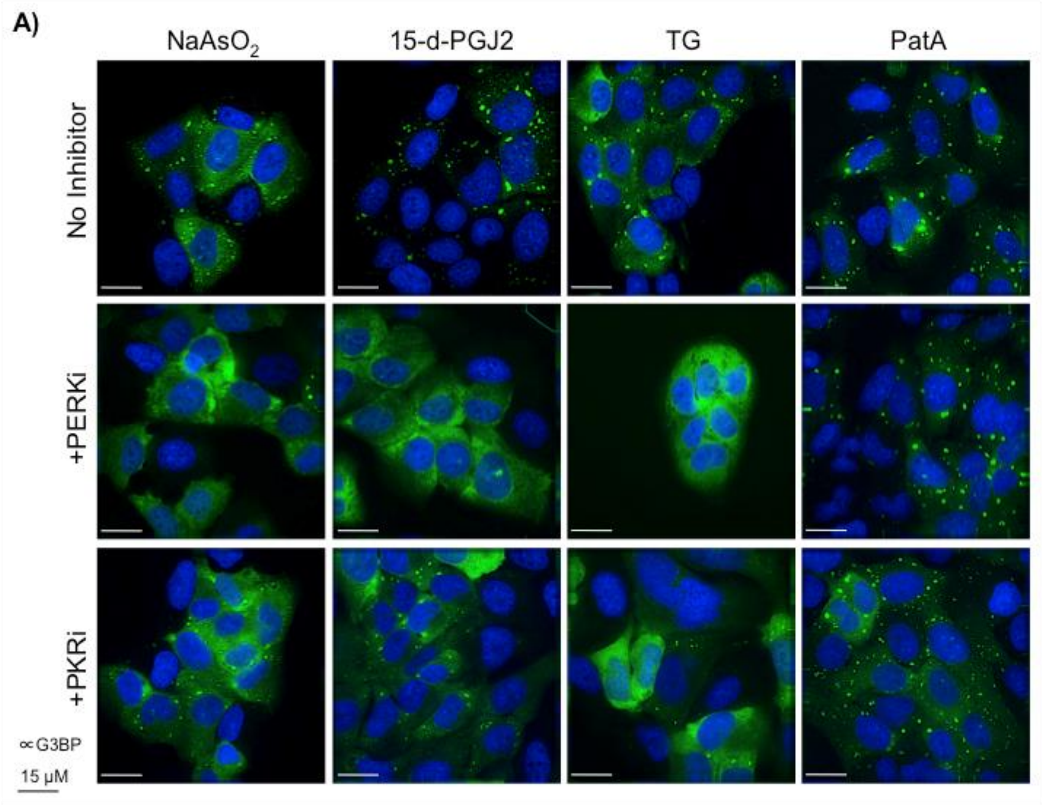

Supplemental Figure 1: eIF2α kinase inhibitors prevent SG formation for 15-d-PGJ2. (**A**) Representative images for the quantifications in Figure 3B&C. The SG response of GFP-G3BP1 expressing U2OS cells for $NaAsO_2$ (100 μM), 15-d-PGJ2 (10 μM), and TG (500 nM) were all diminished with 15 mins pre-incubation with PERKi (1 μM), and the SG responses where slightly diminished with the same pre-incubation in PKRi (1 μM). PatA (100 nM) SGs remained unaffected with inhibitor treatments.

A)

HSPs
Proteasome
Translation
Mitochondria
Negative feedback
Other

Protein	MASCOT Score
HSP90-beta	673
HSP90-alpha	441
Ribonuclease Inhibitor	199
eiF3F	163
Arginyl-tRNA synthetase	158
ATP Synthase	133
Alanyl-tRNA Synthetase	128
Cytochrome b	128
HSP75	121
26S regulatory subunit 2	107
26S regulatory subunit 12	94
26S regulatory subunit 11	94
eiF4A	87
26S regulatory subunit 8	87
PABP1	81
PABP4	78
FMR1 Interacting Protein	77
eiF3D	76
26S regulatory subunit 6B	72
26S regulatory subunit 7	71
60S RP P0	70
26S regulatory subunit 6A	70
PP1	68
26S regulatory subunit 6	65
SRPR	61
GRP75	60
26S regulatory subunit 3	53
eiF4G	52
26S regulatory subunit 13	50
eiF3B	50
26S regulatory subunit 14	49
eiF3H	46
26S regulatory subunit 1	43
DHX9	42
26S regulatory subunit 10	42
PABP3	38

Supplemental Figure 2: 15-d-PGJ2 covalently modifies many protein candidates for eIF2α phosphorylation or translational shutoff.
(**A**) Mass spectrum protein candidates for eIF2αK activation (data from Marcone et al. 2013). 26S regulatory subunits marked in red are bound by 15-d-PGJ2 and are most likely inhibited. However, many more proteins are outlined that could potentially contribute to kinase activation or translational shutoff such as tRNA synthetases (white), HSPs (green), and the eIF2α phosphatase PP1 (orange).

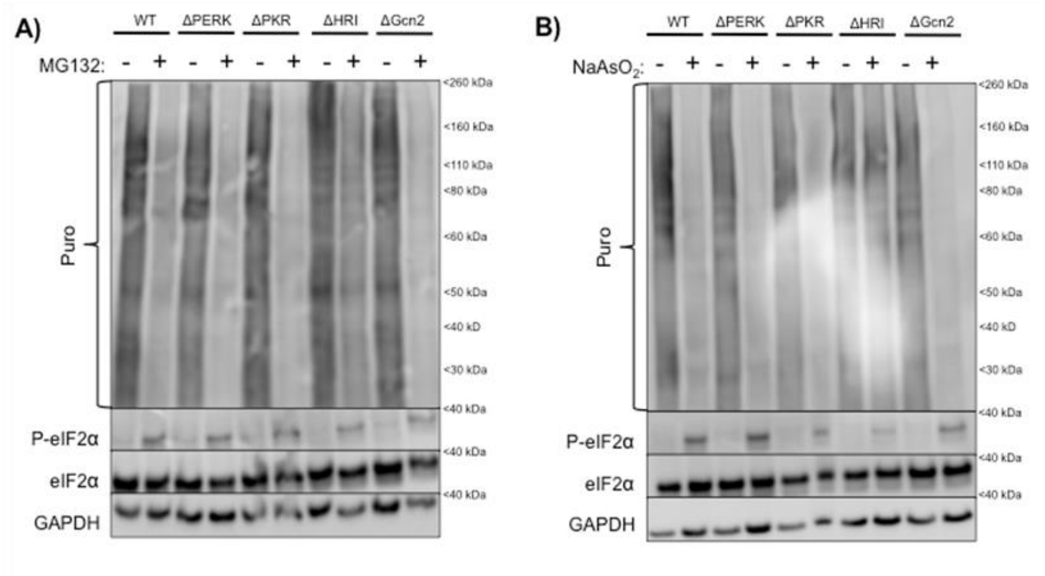

Supplemental Figure 3: MG132 activates multiple eIF2αKs with partial reductions in magnitude from ΔHRI in HAP1 cells.

(**A**) Western blot depicting translation and eIF2α phosphorylation before or after MG132 (10 μM) addition across various kinase deletion backgrounds in HAP1 cells. ΔHRI partially inhibited translational shutoff and P-eIF2α. (**B**) Western Blot depicting $NaAsO_2$ (100 μM) treated HAP1 cells. ΔHRI prevented translational shutoff and partially prevented P-eIF2α.
